# visPedigree: a comprehensive R package for tidying, analyzing, and visualizing breeding pedigrees

**DOI:** 10.1093/bioadv/vbag210

**Published:** 2026-07-29

**Authors:** Sheng Luan, Jie Kong, Zhenglong Xia, Ziyi Kang, Guangfeng Qiang, Kun Luo, Juan Sui

**Affiliations:** State Key Laboratory of Mariculture Biobreeding and Sustainable Goods, Yellow Sea Fisheries Research Institute, Chinese Academy of Fishery Sciences, Qingdao, Shandong 266071, China; State Key Laboratory of Mariculture Biobreeding and Sustainable Goods, Yellow Sea Fisheries Research Institute, Chinese Academy of Fishery Sciences, Qingdao, Shandong 266071, China; Jiangsu Shufeng Prawn Breeding Co., Ltd., Gaoyou 225600, China; State Key Laboratory of Mariculture Biobreeding and Sustainable Goods, Yellow Sea Fisheries Research Institute, Chinese Academy of Fishery Sciences, Qingdao, Shandong 266071, China; State Key Laboratory of Mariculture Biobreeding and Sustainable Goods, Yellow Sea Fisheries Research Institute, Chinese Academy of Fishery Sciences, Qingdao, Shandong 266071, China; State Key Laboratory of Mariculture Biobreeding and Sustainable Goods, Yellow Sea Fisheries Research Institute, Chinese Academy of Fishery Sciences, Qingdao, Shandong 266071, China; State Key Laboratory of Mariculture Biobreeding and Sustainable Goods, Yellow Sea Fisheries Research Institute, Chinese Academy of Fishery Sciences, Qingdao, Shandong 266071, China

## Abstract

**Motivation:**

Pedigrees support relatedness control, mate allocation, inbreeding monitoring, and diversity assessment, but large breeding populations are often curated, analyzed, and visualized using separate tools and data structures. This fragmentation complicates routine analysis and reporting, particularly in high-fecundity systems with extensive full-sib structure, irregular pedigree depth, and repeated cohort-based evaluation.

**Results:**

We present visPedigree, a software package for large breeding pedigrees built around a new tidyped object model. It integrates pedigree standardization, structural validation, candidate-centered tracing, pedigree statistics, inbreeding and partial inbreeding analysis, founder- and ancestor-based diversity summaries, effective population size estimation, relationship matrices, and scalable visualization. The package also provides Shannon/Hill-based diversity measures, the pedhalflife() temporal diagnostic, and compact visualization of full-sib-dominated subsets. In a giant freshwater prawn pedigree, it produced an interpretable ancestry overview and quantified recent diversity erosion. Simulated benchmarks showed that representative workflows completed within seconds for pedigrees containing up to one million individuals on a standard laptop.

**Availability and implementation:**

visPedigree is implemented in the R language and is freely available at https://cran.r-project.org/package=visPedigree, with documentation at https://luansheng.github.io/visPedigree/.

## 1 Introduction

Pedigree information is used across human genealogy, medical genetics, forensic genetics, conservation genetics, and breeding programs to describe ancestry and to quantify relatedness. In breeding management, pedigrees remain especially practical because they support mate allocation, inbreeding monitoring, diversity assessment, and routine reporting across repeated selection cycles. Aquaculture, poultry, livestock, and some plant-breeding systems can contain very large numbers of individuals, many full-sib families, irregular pedigree depth, and cohort-based evaluation. These features make both recursive analysis and visualization difficult at breeding-program scale.

Existing R packages address important parts of this problem, including pedigree objects and plotting, inbreeding-related summaries, relationship-matrix construction, and optimum contribution selection (Garbe and Da 2003, Wolak 2012, Sinnwell *et al*. 2014, Wellmann 2019, López-Cortegano 2022, Le Nézet *et al*. 2025). In practice, the same breeding pedigree is often cleaned, traced, analyzed, and plotted in different tools. This repeated conversion can make parent codes inconsistent or accidentally remove ancestors required for recursive analysis. Visualization is particularly difficult in full-sib-dominated pedigrees, where many visually redundant sibling edges can hide the older ancestry that breeders need to inspect, a recurrent feature of family-based aquaculture breeding programs (Gjedrem 2010). Static diversity summaries are also insufficient on their own when managers need to assess how quickly diversity is being lost across cohorts.

To address these needs, we developed visPedigree, an R package centered on a new tidyped object model. The package integrates pedigree standardization and validation, candidate-centered tracing, pedigree analysis, relationship matrices, diversity diagnostics and scalable visualization within a single workflow. We describe the implementation, illustrate its use in a breeding pedigree of the giant freshwater prawn (*Macrobrachium rosenbergii*) with a complementary cattle pedigree example, and summarize its practical scaling on a simulated large-pedigree series.

## 2 Implementation

### 2.1 The tidyped object model

visPedigree is organized around the new tidyped S3 class, which standardizes core pedigree columns and maintains integer pedigree indices (IndNum, SireNum, DamNum) for downstream recursive algorithms. This avoids repeated identifier matching and provides a stable 1…N representation for large pedigrees. The integer indices, rather than the original character labels, define the computational pedigree used by the recursive and matrix routines.

The class also separates valid pedigree structure from ordinary table manipulation. Complete subsets retain the tidyped class and have indices rebuilt in place, whereas row-truncated subsets that remove required ancestors are degraded to ordinary tables. Centering downstream analyses on a validated object reduces repeated preprocessing and helps prevent silent use of incomplete ancestry.

### 2.2 Pedigree curation and structural validation

The workflow begins with tidyped(), which standardizes raw records by renaming the first three columns to Ind, Sire and Dam, converting identifiers to character values, normalizing common missing-parent encodings to NA, removing invalid individual IDs, checking duplicate records, and detecting parent-role or sex conflicts. Parents that are referenced but absent as rows are inserted as missing founders, and the resulting directed pedigree graph is checked for cycles before analysis proceeds. tidyped() then assigns generation numbers, sorts rows by generation and individual label, rebuilds IndNum, SireNum and DamNum, and attaches pedigree metadata. Because individual labels are stored as character values, ties within a generation are ordered lexicographically; e.g. label “10” precedes “2.” This ordering affects display order only: the recursive pedigree algorithms use the rebuilt integer indices.

Candidate-centered tracing can be performed during object construction or downstream analysis, allowing users to focus on the ancestry of a current cohort or query set while keeping required ancestors. Complete subsets retain the tidyped class and have their indices rebuilt, whereas row-truncated subsets that remove required parents are downgraded to ordinary tables. splitped() further partitions disconnected components when breeding records contain multiple sub-populations. Up-front validation reduces repeated data cleaning and improves reproducibility of downstream results.

### 2.3 Breeding-oriented pedigree analytics

Once pedigrees are standardized, visPedigree provides an integrated analytical chain covering pedigree depth, inbreeding, relationships, diversity and matrices. pedstats() summarizes pedigree depth, completeness and equivalent complete generations, and pedgenint() estimates generation intervals.

inbreed() computes individual coefficients using the recursive algorithm of Sargolzaei *et al*. (2005), which extends the Meuwissen and Luo (1992) path-collapsing strategy for large pedigrees. pedpartial() decomposes inbreeding into ancestor-specific contributions using local path tracing and ancestor-origin propagation on integer-indexed pedigrees. pedcontrib(), pediv(), and pedne() summarize founder and ancestor contributions, effective founder ($f_{e}$), and ancestor ($f_{a}$) numbers (Boichard *et al*. 1997), founder genome equivalents ($f_{g}$; Lacy 1989) and effective population size ($N_{e}$; Caballero and Toro 2000), while pedrel() summarizes relationship or coancestry within and across cohorts. For matrix-based workflows, pedmat() constructs additive (A), dominance (D), and additive-by-additive epistatic (AA) relationship matrices and their inverses, using Henderson’s rules for $A^{-1}$ (Henderson 1976). For analyses that require products rather than individual matrix entries, the new pedprod() function applies A or $A^{-1}$ to a vector or matrix directly from the pedigree. Products with A use the $A=TDT^{'}$ factorization and forward-backward pedigree traversals following Colleau’s indirect approach (Colleau 2002), whereas products with $A^{-1}$ use the sparse inverse structure of the pedigree relationship matrix. Both avoid materializing the dense n×n additive relationship matrix.

### 2.4 Diversity extensions for breeding management

Beyond classical pedigree summaries, visPedigree implements two extensions for diversity interpretation. First, it provides Shannon/Hill-based effective founder ($f_{eH}$) and ancestor ($f_{aH}$) measures, which describe contribution evenness on an effective-number scale (Hill 1973, Jost 2006). Second, pedhalflife() summarizes temporal erosion of founder-genome diversity by following the decomposition


$$\ln f_{g}(t)=\ln f_{e}(t)+\ln(\frac{f_{a}(t)}{f_{e}(t)})+\ln(\frac{f_{g}(t)}{f_{a}(t)}).$$

By fitting linear trends on the log scale, the method estimates component decay rates for founder loss, bottleneck transmission and drift, denoted $\lambda_{e}$, $\lambda_{b}$, and $\lambda_{d}$, together with


$$\lambda_{\mathrm{total}}=\lambda_{e}+\lambda_{b}+\lambda_{d},$$

and a diversity half-life,


$$T_{1/2}=\frac{\ln2}{\lambda_{\mathrm{total}}},$$

where $\lambda_{e}$ captures loss attributable to uneven founder representation, $\lambda_{b}$ captures bottleneck transmission between founders and influential ancestors, and $\lambda_{d}$ captures subsequent drift within the retained pedigree.

### 2.5 Scalable visualization and computational architecture

To address the full-sib visualization challenge, visPedigree combines candidate-centered extraction, topological generation reassignment and compact family-aware layout. When compact = TRUE, terminal full-sib individuals that are not themselves parents can be collapsed into a family display node. This is a display abstraction only: the underlying tidyped pedigree remains an individual-level table. In the default drawing (shapeby = “sex”), individual sex is encoded by shape: females are circles, males are squares, individuals of unknown sex are diamonds, and monoecious individuals are hexagons. Compact full-sib summaries are drawn as separate rectangular FS × *N* nodes, where *N* denotes family size rather than an individual identifier.

visped() represents a pedigree as a layered directed graph with virtual family nodes connecting parents and offspring. It uses igraph’s Sugiyama layout as the hierarchical backbone and then applies pedigree-specific post-processing to center families within generations and reduce horizontal overlaps. Edges are drawn before nodes and labels, which helps preserve visual alignment in vector output. This combination of hierarchical layout and pedigree-specific adjustment supports compact visualization of large traced subsets with many full-sib families.

Implementation combines data.table (Barrett *et al*. 2026) for pedigree manipulation, igraph (Csárdi and Nepusz 2006, Antonov *et al*. 2023) for graph traversal and layered layout, and Rcpp/RcppArmadillo (Eddelbuettel and François 2011, Eddelbuettel and Sanderson 2014) for computational kernels such as inbreeding calculation, ancestry propagation and matrix routines. For analytical scalability in full-sib-dominated pedigrees, pedmat() can use representative-based compact matrix workflows that reduce redundant matrix dimensions while preserving family-level information, whereas pedrel() computes grouped relationship summaries through matrix-free pedigree products.

## 3 Application

We applied visPedigree to pedigree records from the giant freshwater prawn (*Macrobrachium rosenbergii*) nucleus breeding population of Jiangsu Shufeng Prawn Breeding Co., Ltd. The data were analyzed with permission from the company, but individual-level pedigree records are not publicly released because of commercial confidentiality. Quantitative summaries were obtained from a traced pedigree rooted in the latest breeding cohort, so the reported diversity measures describe the ancestry retained in the contemporary reference population.

Note 1, available as supplementary data at *Bioinformatics Advances* online presents a complementary livestock application using ExamplePed, a publicly distributed demonstration subset of the Hinterwald cattle pedigree from the optiSel package (Wellmann 2019).

### 3.1 Pedigree scale and structure

The analyzed pedigree spanned birth years 2015–2024 and included nine consecutive annual breeding cohorts from 2016 to 2024, together with the recorded parents required for recursive analysis. The traced pedigree contained 18 465 individuals, including 141 founders, 561 sires, and 744 dams. After standardization with tidyped(), the pedigree reached a maximum assigned generation number of 10, with a mean equivalent complete generation depth of 7.43, a maximum of 8.89 and an estimated mean generation interval of 1.00 year.

### 3.2 Inbreeding and diversity status of the current breeding cohort

For the 2024 reference cohort, mean inbreeding was 0.0283 and the maximum individual value was 0.3794. Most non-zero inbreeding remained moderate, with 89.52% of inbred individuals having 0<F≤0.0625. Diversity decomposition showed $f_{eH}=96.51$, $f_{e}=78.58$, $f_{aH}=80.75$, $f_{a}=67.12$, $f_{g}=22.74$, and a coancestry-based effective population size of $N_{e}=170.3$. The higher Hill-based values indicate that founder and ancestor contributions retained more evenness than suggested by the classical $f_{e}$ and $f_{a}$ summaries alone, whereas the much lower $f_{g}$ indicates that bottlenecks and drift had already eroded founder-genome diversity more severely.

### 3.3 Temporal diversity erosion

**Figure 1:**
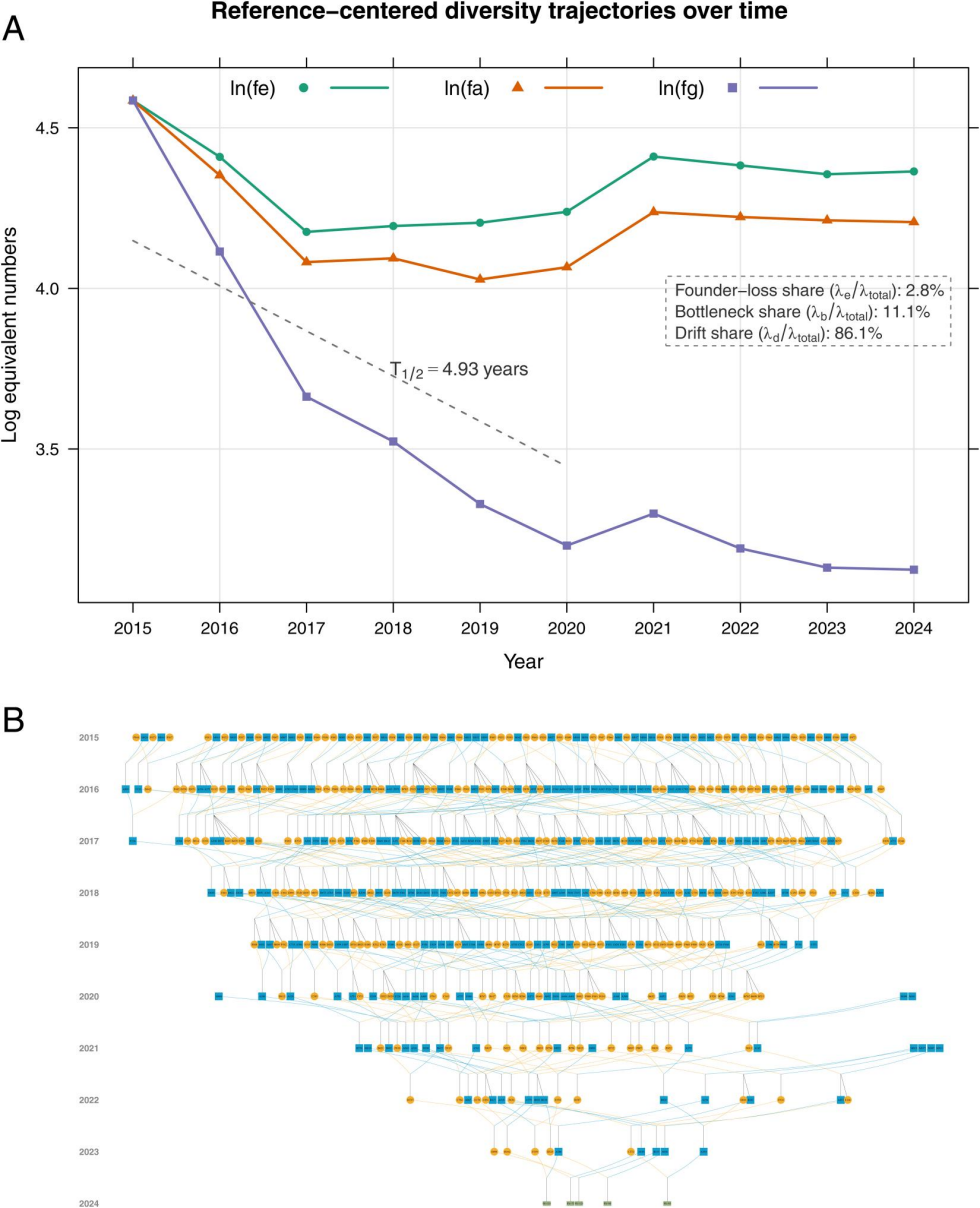
Breeding-oriented application of visPedigree to a large aquaculture pedigree. (A) Log-scale temporal diversity-diagnostic summary obtained with pedhalflife() for the ancestry retained in the 2015–2024 contemporary breeding line. The trajectories of $\ln(f_{e})$, $\ln(f_{a})$, and $\ln(f_{g})$ show cumulative diversity decay within the traced reference-centered pedigree, and the accompanying annotation reports the estimated diversity half-life together with the contributions of founder loss, bottleneck transmission and drift. (B) Candidate-centered compact visualization of an illustrative subset obtained by selecting five families from the latest generation of the giant freshwater prawn breeding population and tracing their recorded ancestors. Individual symbols encode sex by shape in the default plot, whereas rectangular FS × *N* nodes represent compacted terminal full-sib families labeled by the number of represented siblings. The panel is intended as an overview-level diagnostic rather than a node-by-node pedigree diagram, helping reveal generation depth, founder or immigrant entry points and recent family concentration while reducing redundant sibling structure.

Applying pedhalflife() to the traced pedigree across the 2015–2024 time series yielded $\lambda_{\mathrm{total}}=0.1406$, corresponding to a fitted diversity half-life of 4.93 years within the ancestry retained by the 2024 reference cohort. The fitted component rates for founder loss, bottleneck transmission and drift (Fig. 1A) were $\lambda_{e}=0.0039$, $\lambda_{b}=0.0156$, and $\lambda_{d}=0.1211$, corresponding to relative contributions of 2.8%, 11.1%, and 86.1%, respectively. Thus, the recent decline was driven mainly by drift rather than by founder loss, suggesting that diversity management should prioritize balancing family contributions within each cohort. Given a generation interval of approximately one year, the fitted half-life corresponds to roughly five breeding cycles. Over the same period, $f_{g}$ declined from 98.0 to 22.7, a 76.8% reduction within the retained ancestry of the contemporary cohort.

### 3.4 Visualization of a candidate-centered breeding pedigree subset

To demonstrate diagnostic visualization in a real breeding pedigree, we selected five families from the latest generation and traced all recorded ancestors of the corresponding individuals. The compact view provides an overview of the ancestry retained by these recent families, including pedigree depth, founder or unrecorded-parent entry points and the degree to which current families are concentrated through particular ancestral branches. This view supports routine breeding review by making recent family structure and historical ancestry inspectable without displaying every terminal sibling separately (Fig. 1B).

### 3.5 Scalability on simulated large pedigrees

**Figure 2:**
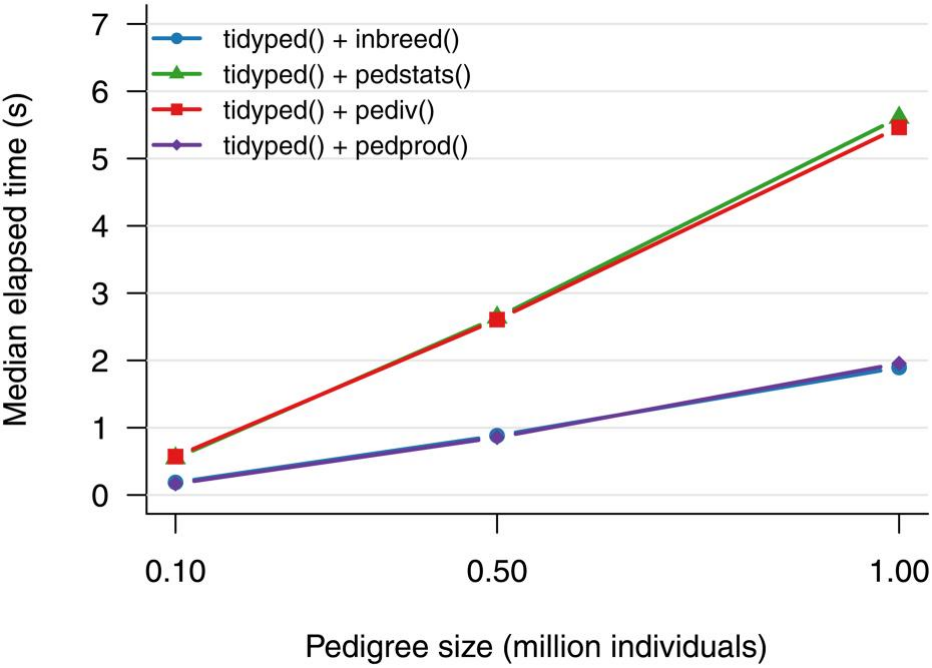
Scalability of representative visPedigree workflows on simulated large pedigrees. Elapsed time is shown for four end-to-end workflows, tidyped() + inbreed(), tidyped() + pedstats(), tidyped() + pediv(), and matrix-free tidyped() + pedprod(), across cumulative simulated pedigree subsets containing N=100 150, 500 150, and 1 000 150 individuals. The simulated pedigree contained 150 founders and 20 000 additional individuals per generation through generation 50. Benchmarks were run on macOS 26.3 (Apple M2, 16 GB RAM) under R 4.5.2. Full workflow definitions and values are provided in Table 2, available as supplementary data at *Bioinformatics Advances* online.

To provide reproducible scaling evidence, we benchmarked four representative workflows on cumulative subsets of a simulated pedigree with 150 founders and 20 000 individuals per generation through generation 50. The tested subsets contained *N* = 100 150, 500 150, and 1 000 150 individuals, and the workflows were tidyped() + inbreed(), tidyped() + pedstats(), tidyped() + pediv(), and tidyped() + pedprod() (Fig. 2; Table 2, available as supplementary data at *Bioinformatics Advances* online). On macOS 26.3 (Apple M2, 16 GB RAM, R 4.5.2), the workflows completed in 0.19–1.90 s, 0.55–5.61 s, 0.57–5.46 s, and 0.17–1.96 s, respectively.

## 4 Discussion

In industrial breeding programs, pedigree records are large, continuously updated and repeatedly reused for curation, candidate tracing, cohort summaries, inbreeding and diversity analysis, relationship-matrix construction, and diagnostic visualization. visPedigree addresses this routine workflow by linking these steps through the tidyped object model, which keeps identifiers, generation assignments, integer pedigree indices, structural metadata and downstream analyses aligned within one validated framework.

### 4.1 Position relative to existing software

Existing pedigree software differs in domain emphasis. kinship2 and Pedixplorer provide pedigree object handling, kinship or identity-by-descent structures and family-pedigree visualization, with strong relevance to medical and family-based applications. nadiv focuses on additive and non-additive genetic relatedness matrices and their inverses for animal-model analyses, whereas optiSel supports optimum contribution selection, mate allocation and related population-genetic evaluations. purgeR emphasizes inbreeding-purging analysis, including purged inbreeding coefficients and inbreeding-load-related measures. These packages remain valuable for their specialist purposes.

The distinction of visPedigree is that it organizes the main steps of large breeding-pedigree analysis within a single tidyped-centered workflow, from curation, validation and candidate-centered tracing to inbreeding analysis, relationship matrices, diversity summaries and visualization. Several components are designed specifically for large pedigrees, including matrix-free relationship products through pedprod(), compact full-sib visualization with explicit family-summary nodes and diversity-oriented extensions such as Shannon/Hill-based effective founder and ancestor measures and the temporal diagnostic pedhalflife(). These components reduce repeated preprocessing, preserve integer pedigree indices for recursive algorithms, avoid dense relationship matrices when only products are required and reduce redundant full-sib structure in plots. In the benchmark, representative workflows completed within seconds for simulated pedigrees containing up to one million individuals.

Interoperability is supported at the standard tabular pedigree level. tidyped() accepts ordinary data.frame or data.table inputs whose first three columns contain individual, sire and dam identifiers, so pedigrees exported from other software can enter the workflow after conversion to this common ID-sire-dam format. The current version does not provide native converters for every external S3 or S4 pedigree object class, because such converters require package-specific assumptions and testing. A broader feature-chain comparison is provided in the Supplementary Material, available as supplementary data at *Bioinformatics Advances* online. Overall, visPedigree should be viewed as a scalable workflow complement for large breeding pedigrees rather than as a replacement for every specialist pedigree-analysis package.

### 4.2 Practical scalability

The benchmark results indicate that common visPedigree workflows can scale to million-individual pedigrees on a standard laptop. Scalability nevertheless depends on the requested output. Dense relationship matrices remain quadratic in memory, whereas pedprod(), compact full-sib workflows and reference-centered subsets provide more scalable alternatives when users need matrix products, summaries or selected pedigree views.

### 4.3 Management interpretation and limitations

From a breeding-management perspective, the classical summaries F, $f_{e}$, $f_{a}$, $f_{g}$, and $N_{e}$ mainly describe current genetic status. visPedigree adds three management-oriented diagnostic quantities: the Shannon/Hill-based founder and ancestor measures $f_{eH}$ and $f_{aH}$, and the temporal diversity diagnostic pedhalflife(). These additions are intended to make pedigree diversity summaries easier to interpret for selection and mating decisions. Lower $f_{eH}$ suggests uneven retention of founder-derived lineages and therefore supports retaining candidates from under-represented origins. Lower $f_{aH}$ indicates concentration in a limited set of influential ancestors, which can help identify recent bottlenecks and discourage repeated use of closely related high-contribution lines. A short diversity half-life indicates rapid erosion of retained diversity, and its component decomposition helps distinguish whether management should prioritize restoring poorly represented founder origins, reducing ancestor bottlenecks or equalizing family contributions across the next round of selection and mating.

The package also has clear limitations. It is pedigree-based rather than genomic, so it does not incorporate marker data or combined pedigree-genomic relationship matrices. Traced-pedigree results depend on the chosen reference cohort, and compact visualization is a display abstraction rather than a change to pedigree structure. Although the examples emphasize breeding programs, the workflow can also be useful for other large pedigrees that can be represented as parent-offspring tables. Conversely, small clinical pedigrees requiring full medical-pedigree diagram conventions and disease-annotation semantics are not the default design target. The diversity half-life framework is a management-oriented trend summary, not a full stochastic population-genetic model.

## 5 Conclusion

visPedigree provides a unified pedigree workflow for breeding populations and other large tabular pedigrees, linking curation, structural validation, recursive analysis, relationship matrices and scalable visualization. By centering these tasks on a validated pedigree object, it makes large pedigree analysis more coherent and extends routine summaries with diversity-oriented diagnostics.

## Supplementary Material

vbag210_Supplementary_Data

## Data Availability

The visPedigree software is available from the Comprehensive R Archive Network at https://cran.r-project.org/package=visPedigree, with documentation at https://luansheng.github.io/visPedigree/. The simulated benchmark pedigree, benchmark script, raw timing data, median summaries and run metadata are available from Zenodo at https://doi.org/10.5281/zenodo.21320956. The Hinterwald cattle workflow and generated SVG are available from Zenodo at https://doi.org/10.5281/zenodo.21321031. Individual-level pedigree records from the giant freshwater prawn breeding population are not publicly available because of commercial confidentiality.
