# Supplementary material for: visPedigree: a comprehensive R package for tidying, analyzing, and visualizing breeding pedigrees
Source: Bioinform Adv. 2026 Jul 29;6(1):vbag210. doi: 10.1093/bioadv/vbag210 (PMC13479741; doi:10.1093/bioadv/vbag210)
Supplement: Supplementary file 1 [file vbag210_supplementary_data.pdf]

Supplementary materials

Supplementary Table S1. Feature-chain comparison of representative pedigree software

Workflow component	visPedigree	Pedixplorer	purgeR	optiSel	nadiv	kinship2
Pedigree standardization / validation	**Yes**	Partial	Partial	Partial	Partial	Partial
Candidate-centered tracing / trimming	**Yes**	Yes	No	Partial	Partial	Yes
Inbreeding coefficients	**Yes**	Partial	Yes	Yes	Partial	Partial
Exact partial inbreeding	**Yes**	No	Yes	No	No	No
Founder / ancestor diversity (f_e, f_a, f_g)	**Yes**	No	Yes	Partial	No	No
Effective population size summaries	**Yes**	No	Yes	Yes	No	No
Relationship summaries / kinship	**Yes**	Partial	No	Yes	No	Yes
Relationship matrices (A / A^{-1} or non-additive variants)	**Yes**	No	No	Partial	Yes	No
Pedigree plotting / visualization	**Yes**	Yes	No	Partial	No	Yes
Explicit full-sib compaction for display	**Yes**	No	No	No	No	No
Temporal diversity diagnostics	**Yes**	No	No	No	No	No

Notes for Supplementary Table S1

The table summarizes workflow emphasis rather than ranking packages. Coding was kept conservative and based on documented user-facing functionality. “Yes” indicates that the component is directly exposed as a documented user workflow; “Partial” indicates narrower support, indirect derivation, or infrastructure from which the quantity can be obtained but is not presented as a central end-user feature; “No” indicates that we did not find documented support for that component. For nadiv, inbreeding coefficients are returned as a by-product of makeDiiF(). For kinship2 and Pedixplorer, inbreeding can be derived from the kinship matrix diagonal. For purgeR, pedigree preprocessing functions support partial standardization, and pop_Nancestors() provides effective founder, ancestor and founder-genome metrics. For nadiv, prepPed() and prunePed() support partial preprocessing and trimming. For optiSel, subPed() supports individual-centered sub-pedigree extraction. The comparison is intended to clarify domain emphasis: for example, kinship2 and Pedixplorer emphasize pedigree object handling and display, nadiv emphasizes relationship matrices, optiSel emphasizes optimum contribution selection, purgeR emphasizes purging and inbreeding-load analyses, and visPedigree emphasizes an integrated tidyped workflow for large breeding-oriented pedigrees.

Supplementary Note S1. Hinterwald cattle pedigree workflow

We analyzed ExamplePed, a publicly distributed demonstration subset of the Hinterwald cattle pedigree from the optiSel package (Wellmann, 2019). The dataset contains 1,179 records with individual, sire, dam, sex, breed and birth-year fields.

The complete reproducible workflow and generated SVG are archived at Zenodo: https://doi.org/10.5281/zenodo.21321031. It standardizes the pedigree with tidyped(), summarizes pedigree structure with pedstats(), applies the matrix-free pedprod() workflow to an equally weighted latest-year candidate group, and writes the candidate-centred SVG used below.

The standardized pedigree contained 330 founders and extended to generation 14; 292 animals had non-zero inbreeding, with mean F = 0.0037. The mean generation interval was 5.43 years and the mean equivalent complete generations value was 2.36. The latest birth year (1995) contained 10 animals. For these equally weighted candidates, the mean additive relationship was c'Ac = 0.1461, corresponding to a mean coancestry of 0.0730. Restricting the display to two to four ancestral generations produced a 59-record pedigree.

Supplementary Figure S1. Candidate-centred Hinterwald cattle pedigree

The sex-coded plot highlights the 10 animals in the 1995 candidate group and shows their two to four traced ancestral generations.

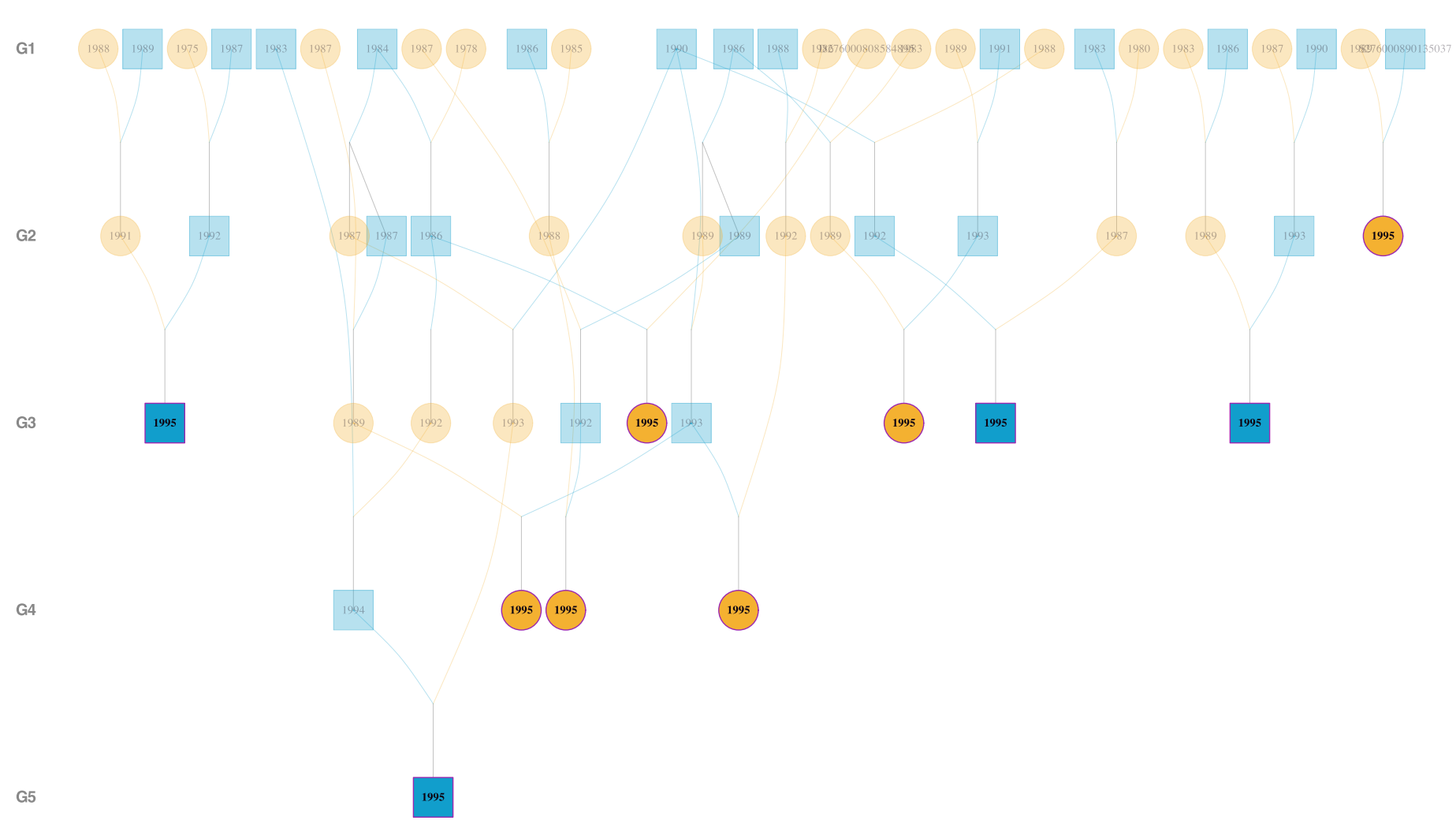

Figure S1. Candidate-centred Hinterwald cattle pedigree.

Supplementary Table S2. Scalability of representative visPedigree workflows

This table reports scalability results for representative visPedigree workflows on a unified simulated pedigree series. The same values are summarized as a runtime line plot in Fig. 2 of the main text.

Workflow definition

- tidyped() + inbreed(): end-to-end pedigree preprocessing and recursive inbreeding analysis
- tidyped() + pedstats(): pedigree preprocessing and structural summary (timevar = “Year”, ecg = TRUE, genint = TRUE)
- tidyped() + pediv(): pedigree preprocessing and diversity analysis (reference = latest cohort, nsamples = 1000, seed = 42)
- tidyped() + pedprod(): pedigree preprocessing followed by matrix-free multiplication of A by an equal-contribution vector for the 20,000 individuals in the latest cohort

Benchmark dataset and test environment

All scales were derived from the same simulated pedigree dataset, archived at https://doi.org/10.5281/zenodo.21320956, with 150 founders at generation 0 and 20,000 additional individuals per generation through generation 50. Benchmarks were run on macOS 26.3 (Apple M2, 16 GB RAM) under R 4.5.2. Cumulative subsets were defined as follows:

- 100k scale: generations 0–5 (N = 100,150)
- 500k scale: generations 0–25 (N = 500,150)
- 1M scale: generations 0–50 (N = 1,000,150)

Table S2. Unified simulated-pedigree benchmark

Workflow	100k (N = 100,150)	500k (N = 500,150)	1M (N = 1,000,150)
tidyped() + inbreed()	0.19 s	0.88 s	1.90 s
tidyped() + pedstats()	0.55 s	2.64 s	5.61 s
tidyped() + pediv()	0.57 s	2.61 s	5.46 s
tidyped() + pedprod()	0.17 s	0.86 s	1.96 s

Notes for Supplementary Table S2

1. Each complete visPedigree workflow was first warmed up once and then timed five times with system.time() on the same machine and source pedigree series. Reported values are the median elapsed times across the five timed repetitions; garbage collection was run immediately before every warm-up and timed repetition.
2. The benchmark script (benchmark_simulated_scales.R), raw timing data, median summaries, and run metadata are available at Zenodo: https://doi.org/10.5281/zenodo.21320956
